# Results of peripheral bypass surgery in patients with critical limb ischemia (CRITISCH registry)

**DOI:** 10.1007/s00772-016-0166-2

**Published:** 2016-07-13

**Authors:** T. Bisdas, G. Torsello, A. Stachmann, R. T. Grundmann, CRITISCH study group

**Affiliations:** 1 Klinik für Vaskuläre und Endovaskuläre Chirurgie, Universitätsklinikum and St. Franziskus Hospital Münster, Münster, Germany; 2 Deutsches Institut für Gefäßmedizinische Gesundheitsforschung (DIGG) of the DGG, Berlin, Germany; 3 Wiss. Koordinator DIGG, In den Grüben 144, 84489 Burghausen, Germany

**Keywords:** Critical limb ischemia, Bypass surgery, Autologous vein, Prosthetic graft, Amputation-free survival, Kritische Extremitätenischämie, Bypasschirurgie, Autologe Vene, Gefäßprothese, Amputationsfreies Überleben

## Abstract

**Aim:**

On the basis of the CRITISCH registry outcomes in patients with critical limb ischemia (CLI) undergoing lower extremity bypass surgery were analyzed according to the site of distal anastomosis and type of bypass material.

**Patients and methods:**

A total of 284 patients with lower extremity bypasses consisting of 75 patients with bypasses above the knee (group 1), 80 with bypasses below the knee (group 2) and 129 crural or pedal bypasses (group 3) were included in the study. Altogether, 159 autologous saphenous vein grafts and 125 synthetic grafts were used.

**Results:**

There were no perioperative complications in 191 out of the 284 patients (67.3 %) and 236 of the 284 patients (83.1 %) had open bypasses at hospital discharge. An uneventful postoperative course was observed in 76 % of the patients in group 1, 62.5 % in group 2 and 65.1 % in group 3. Amputation-free survival was 86 % at 1 year in group 1, 65 % in group 2 and 69 % in group 3. For bypasses above the knee synthetic grafts were at least not inferior to vein grafts (amputation-free survival at 1 year: prosthetic bypasses 92 % and saphenous vein grafts 71 %, *p* = 0.147), whereas in the crural/pedal bypass group vein grafts showed better amputation-free survival at 1 year (76 %) compared with synthetic bypasses (56 %, *p* = 0.105). Patients with a PREVENT III (PIII) CLI risk score ≤3 exhibited better amputation-free survival at 1 year (78 %) compared to patients with a PIII CLI risk score of 4–7 (69 %, *p* = 0.053). The same applied to patients with Rutherford class 4 vs. Rutherford class 6 CLI.

**Conclusion:**

In patients with CLI and above-knee bypasses, vein grafts confer no benefits compared with synthetic grafts for at least 1 year follow-up; however, in the case of more distal anastomoses vein grafts should be preferred.

## Zusammenfassung

**Zielsetzung:**

Die Ergebnisse der peripheren Bypasschirurgie in Abhängigkeit von der Lokalisation des distalen Anschlusses und von der Art des Bypassmaterials werden anhand der Daten des CRITISCH-Registers bei Patienten mit kritischer Extremitätenischämie (CLI) dargestellt.

**Patienten und Methodik:**

Insgesamt 284 CLI-Patienten mit peripherem Bypass (75 Patienten mit Bypässen oberhalb des Knies [Gruppe 1], 80 Patienten mit Bypässen unterhalb des Knies [Gruppe 2] und 129 krurale/pedale Bypässe [Gruppe 3]) wurden analysiert. 159 Venenbypässe standen 125 Kunststoffbypässen gegenüber.

**Ergebnisse:**

Im Gesamtkrankengut verlief der Eingriff bei 191 von 284 Patienten (67,3 %) ohne Komplikationen, bei 236 von 284 (83,1 %) Patienten war der Bypass bei Entlassung offen. 76 % der Patienten in Gruppe 1, 62,5 % in Gruppe 2 und 65,1 % in Gruppe 3 zeigten einen komplikationslosen postoperativen Verlauf. Das amputationsfreie Überleben betrug nach 1 Jahr 86 % in Gruppe 1, 65 % in Gruppe 2 und 69 % in Gruppe 3. Bei Bypässen oberhalb des Kniegelenks war Kunststoffmaterial dem Venenbypass zumindest nicht unterlegen (amputationsfreies Überleben nach 1 Jahr: Kunststoffbypass 92 %, Venenbypass 71 %, *p* = 0,147), während umgekehrt in der Gruppe der kruralen/pedalen Bypässe Venenbypässe mit 76 % ein besseres amputationsfreies Überleben nach 1 Jahr zeigten als Kunststoffbypässe mit 56 % (*p* = 0,105). Patienten mit einem PREVENT-III(PIII)-CLI-Risikokscore ≤3 wiesen mit 78 % ein besseres amputationsfreies Überleben nach 1 Jahr auf als Patienten mit PIII-CLI-Risikoscore 4–7 (69 %; *p* = 0,053). Das Gleiche galt für Patienten mit Stadium 4 vs. Stadium 6 nach Rutherford.

**Schlussfolgerung:**

Bei Patienten mit CLI im Follow-up über 1 Jahr bietet es keine Vorteile für Bypässe mit Anschlüssen oberhalb des Knies, Venen statt Kunststoffimplantate zu verwenden, wohl aber bei weiter distal gelegenen Anastomosen.

It is common guideline practice, also according to the American Heart Association (AHA) [1], to recommend the use of autologous veins for peripheral bypass surgery in patients with critical limb ischemia (CLI) wherever possible. In the absence of a suitable vein other materials, such as synthetic prostheses need to be used. In such cases the position of the distal anastomosis plays a significant role in terms of the prospects of success. Whereas synthetic materials can be effectively used for bypasses to the popliteal artery below the knee, according to the guidelines a prosthetic femorotibial bypass should only be used when amputation is imminent and the prospects of success are limited. On the other hand, out of 1227 patients in the registry of the Vascular Study Group of New England for 2003–2006 undergoing below-knee bypasses for CLI, 223 patients (18.2 %) received a prosthetic graft [2]. Of these bypasses 70 % were to the popliteal artery and 30 % to a more distal target. Patients receiving prosthetic grafts were treated for anticoagulation with warfarin more frequently (57 %) compared with those receiving vein bypasses (24 %). Following risk adjustment no significant differences in outcome were seen between prosthetic and vein bypasses (primary patency rate at 1 year 72 % vs. 73 % and major amputation rate 17 % vs. 13 %, respectively), thus indicating the validity of the prosthetic bypass in the absence of a suitable vein and with appropriate anticoagulation. The present study examined the results of peripheral bypass surgery according to (a) the site of distal anastomosis and (b) the type of bypass material by analyzing the data of the CRITISCH registry [3, 4].

## Patients and methods

The methodology of the CRITISCH study has been described elsewhere [3, 4]. A diagnosis of CLI symptoms of more than 2 weeks standing was the criterion for inclusion in the CRITISCH registry and CLI was defined as peripheral arterial occlusive disease and an ankle/brachial index of ≤ 0.4 and/or residual pain (Rutherford class 4 or Fontaine stage III) and/or trophic disorders or necrosis/gangrene in the lower extremities (Rutherford classes 5/6 or Fontaine stage IV). Study endpoints were as follows:

Primary efficacy:

1. Amputation above the ankle of the index extremity or death from any cause, whichever occurred first.

Secondary endpoints:

1. Perioperative death within 30 days.
2. Major adverse limb event (MALE) in the index extremity at 1 and 2 years, such as amputation above the ankle or major intervention (new bypass, bypass revision (jump/interposition) or thrombectomy/thrombolysis).
3. Major adverse cardiovascular event (MACE): myocardial infarction, stroke or death from any cause.
4. Sustained clinical improvement at 2 years: upward shift on the Rutherford or Fontaine classification to the level of intermittent claudication in amputation-free surviving patients not requiring (primary improvement) or following (secondary improvement) repeat target lesion revascularization.
5. Any reintervention or amputation above the ankle in the index limb at 2 years, where reintervention was defined as any repeated vascular intervention in the index limb.
6. Any reintervention, amputation above the ankle in the index limb or stenosis/occlusion at 2 years.
7. Overall survival at 2 years.

Between January 2013 and September 2014 a total of 1200 CLI patients were prospectively included in the registry and 284 underwent peripheral bypass surgery. Of these 284 patients 75 underwent above-knee bypasses (group 1), 80 below-knee bypasses (group 2) and 129 crural/pedal bypasses (group 3). A total of 159 vein bypasses were performed compared with 125 synthetic bypasses. Autologous vein bypasses in groups 1, 2 and 3 were carried out in 36 %, 57.5 % and 66.7 %, respectively. The total patient population comprised 192 males and 92 females. Tables 1 and 2 provide patient age and gender, comorbidities and baseline findings for the three groups.

**Table 1:** Patient characteristics of 284 patients receiving peripheral bypasses for critical limb ischemia

Pre-existing diseases/risk factors	Group 1 (*n* = 75)	Group 2 (*n* = 80)	Group 3 (*n* = 129)
Number of men (%)	52 (69.3)	47 (58.8)	93 (72.1)
Number of women (%)	23 (30.7)	33 (41.2)	36 (27.9)
Patient age (years, mean/min–max)	71.6 (35–91)	71.9 (51–95)	71.8 (45–90)
Age of females (years, mean/min–max)	73.1 (35–88)	77.2 (54–95)	76.9 (49–90)
Age of males (years, mean/min–max)	70.8 (51–91)	68.1 (51–85)	69.8 (45–89)
Patients aged over 80 years, *n* (%)	17 (22.7)	22 (27.5)	27 (20.9)
Angina/coronary heart disease, *n* (%)	33 (44.0)	35 (43.8)	50 (38.8)
History of myocardial infarction (<6 months), *n* (%)	4 (5.3)	8 (10.0)	5 (3.9)
History of stroke/TIA, *n* (%)	11 (14.7)	9 (11.3)	11 (8.5)
Smokers (current), *n* (%)	27 (36.0)	25 (31.3)	37 (28.7)
Diabetes mellitus, *n* (%)	36 (48.0)	30 (37.5)	70 (54.3)
Obesity (BMI >30 kg/m^2^), *n* (%)	10 (13.3)	13 (16.3)	17 (13.2)
GFR 15–29 ml/min/1.73 m^2^, *n* (%)	10 (13.3)	4 (5)	3 (2.3)
GFR <15 ml/min/1.73 m^2^ or dialysis, *n* (%)	3 (4)	3 (3.8)	7 (5.4)
Previous vascular procedure, *n* (%)	27 (36.0)	36 (45)	76 (58.9)
PREVENT III (PIII) CLI risk score ≤3, *n* (%)	35 (46.6)	38 (47.5)	62 (48.1)
PREVENT III (PIII) CLI risk score 4–7, *n* (%)	37 (49.4)	39 (48.7)	62 (48.1)
PREVENT III (PIII) CLI risk score ≥8, *n* (%)	3 (4.0)	3 (3.8)	5 (3.8)

*BMI* body mass index, *CLI* critical limb ischemia, *GFR* glomerular filtration rate, *TIA* transient ischemic attack

**Table 2:** Rutherford classification of critical limb ischemia, peripheral run-off vessels and vein grafts used in groups 1–3

	Group 1 (*n* = 75)	Group 2 (*n* = 80)	Group 3 (*n* = 129)
Rutherford classification			
Class 4, *n* (%)	17 (22.7)	26 (32.5)	29 (22.5)
Class 5, *n* (%)	39 (52.0)	36 (45.0)	65 (50.4)
Class 6, *n* (%)	19 (25.3)	18 (22.5)	35 (27.1)
Run-off, vessels open, *n* (%)			
0, *n* (%)	9 (12.0)	3 (3.7)	19 (14.7)
1, *n* (%)	20 (26.7)	29 (36.3)	85 (65.9)
2, *n* (%)	27 (36.0)	34 (42.5)	15 (11.6)
3, *n* (%)	19 (25.3)	14 (17.5)	10 (7.8)
Vein graft, *n* (%)	27 (36)	46 (57.5)	86 (66.7)

Table 1 also provides the modified PREVENT III (PIII) CLI risk score, designed to predict long-term outcome of infrainguinal vascular reconstruction in CLI patients [5, 6]. Points were assigned to each patient as follows: dialysis 4 points, tissue loss 3 points, age ≥75 years 2 points and coronary artery disease 1 point. The total number of points yields a score on the basis of which patients were stratified into risk categories as low risk (score ≤3), medium risk (score 4–7) and high risk (score ≥8).

### Statistical analysis

The present study was a planned subgroup analysis of the CRITISCH study. Statistical analysis was performed using MedCalc statistical software (version 12.4.0.0, Ostend, Belgium). Descriptive statistics are presented for categorical variables as absolute numbers and percentages and continuous parameters as median (max–min interval). Categorical variables were compared using the χ^2^-test. In a first step, differences in complications between groups were compared. Analysis between patients with and without complications was performed with respect to the effect of the various prospectively recorded risk factors. Complications included the parameters wound infection, lymphatic fistula, compartment syndrome, acute coronary event, stroke/transient ischemic attack (TIA) and major amputation. Amputation-free survival, freedom from amputation, survival and freedom from reinterventions were calculated and plotted using Kaplan-Meier curves. The rates of the abovementioned results were compared between groups using the log-rank test and calculated descriptively for each risk factor. The hazard ratio was presented with a 95 % confidence interval. Differences with *p*-values <0.05 were considered statistically significant.

## Results

### Perioperative results

Tables 3 and 4 show the perioperative results. Looking at the total patient population the procedure was without complications in 191 out of 284 patients (67.3 %) and bypasses were open at the time of hospital discharge in 236 out of 284 patients (83.1 %). No significant differences were observed between the three groups, although the results in group 1 showed a more favorable trend compared with the other groups. This was based on the greater proportion of patients with a complication-free postoperative course in group 1 (76.0 %) vs. groups 2 and 3 (62.5 % and 65.1 %, respectively, *p* = 0.076), the lower number of reinterventions as well as major and in particular, minor amputations and lastly, the approximately 3‑day shorter period of hospitalization (Table 3).

**Table 3:** Postoperative morbidity and mortality in the study groups

	Group 1 (*n* = 75)	Group 2 (*n* = 80)	Group 3 (*n* = 129)
Patients with no complications, *n* (%)	57 (76.0)	50 (62.5)	84 (65.1)
Males with no complications, *n* (%)	39/52 (75.0)	30/47 (63.8)	57/93 (61.3)
Females with no complications, *n* (%)	18/23 (78.3)	20/33 (60.6)	27/36 (75.0)
Bypass open at discharge, *n* (%)	65 (86.6)	67 (83.8)	104 (80.6)
Wound infection, *n* (%)	9 (12.0)	8 (10.0)	14 (10.9)
Lymphatic fistula, *n* (%)	4 (5.3)	7 (8.8)	10 (7.8)
Compartment syndrome, *n* (%)	1 (1.3)	1 (1.3)	3 (2.3)
Reinterventions, *n* (%)	8 (10.7)	7 (8.8)	22 (19.0)
Acute coronary event, *n* (%)	0	3 (3.8)	4 (3.1)
Stroke/TIA, *n* (%)	1 (1.3)	0	0
Major amputation, *n* (%)	1 (1.3)	3 (3.6)	6 (4.7)
Minor amputation, *n* (%)	7 (9.3)	12 (15)	20 (15.5)
Death, *n* (%)	1 (1.3)	3 (3.8)	4 (3.1)
Duration of hospitalization, days (mean/min–max)	18.9 (5–65)	21.5 (6–93)	21.7 (5–63)

*TIA* transient ischemic attack

The parameters wound infection, lymphatic fistula, compartment syndrome, acute coronary event, stroke/TIA, major amputation and death are included under the term complications

**Table 4:** Postoperative outcome according to CLI category, run-off and other risk factors

	Patients without complications	Bypass open at discharge
Males, *n* (%)	126/192 (65.6)	159/192 (82.8)
Females, *n* (%)	65/92 (70.7)	77/92 (83.7)
Patients aged over 80 years, *n* (%)	44/66 (66.7)	57/66 (86.4)
Patients aged less than 80 years, *n* (%)	147/218 (67.4)	179/218 (82.1)
Rutherford class 4, *n* (%)	60/72 (83.3)	64/72 (88.9)
Rutherford class 5, *n* (%)	92/140 (65.7)	122/140 (87.1)
Rutherford class 6, *n* (%)	39/72 (54.2)	50/72 (69.4)
Run-off, vessels open		
0, *n* (%)	19/31 (61.3)	20/31 (64.5)
1, *n* (%)	91/134 (67.9)	115/134 (85.8)
2, *n* (%)	48/76 (63.2)	66/76 (86.8)
3, *n* (%)	33/43 (76.7)	35/43 (81.4)
PIII CLI risk score ≤3, *n* (%)	99/135 (73.3)	117/135 (86.7)
PIII CLI risk score 4–7, *n* (%)	85/138 (61.6)	110/138 (79.7)
PIII CLI risk score ≥8, *n* (%)	7/11 (63.6)	9/11 (81.8)
Diabetics, *n* (%)	83/136 (61.0)	113/136 (83.1)
Non-diabetics, *n* (%)	108/148 (73.0)	123/148 (83.1)
Obesity (BMI >30 kg/m^2^), *n* (%)	26/40 (65.0)	30/40 (75.0)
BMI <30 kg/m^2^, *n* (%)	165/244 (67.6)	206/244 (84.4)
Previous vascular procedure		
Yes, *n* (%)	89/139 (64.0)	112/139 (80.6)
No, *n* (%)	102/145 (70.3)	124/145 (85.5)
Vein graft, *n* (%)	112/159 (70.4)	134/159 (84.3)
Prosthetic graft, *n* (%)	79/125 (63.2)	102/125 (81.6)

*BMI* body mass index, *PIII CLI *PREVENT III critical limb ischemia

Table 4 shows complication-free courses and graft patency rates at discharge according to potential risk factors for the total patient population. Differences were seen for Rutherford class 6 (54.2 % complication-free) vs. Rutherford class 4 (83.3 % complication-free, *p* = 0.0003), no open run-off vessel (61.3 % complication-free) vs. three open run-off vessels (76.7 % complication-free, *p* = 0.239), PIII CLI risk score ≥8 (63.6 % complication-free) vs. PIII CLI risk score ≤3 (73.3 % complication-free, *p* = 0.732) and for diabetics (61.0 % complication-free) vs. non-diabetics (73.0 % complication-free, *p* = 0.044). In terms of graft patency rates at the time of discharge, the greatest differences were seen between Rutherford class 6 (69.4 % open) vs. Rutherford class 4 (88.9 % open, *p* = 0.008) and run-off vessels (no detectable run-off vessel vs. one run-off vessel, bypass open in 64.5 % vs. 85.8 % of cases, *p* = 0.012).

### Follow-up results

Table 5 provides the follow-up results for the three groups. At 86 %, amputation-free survival at 1 year was significantly (*p* = 0.029) better in group 1 compared with group 2 (65 %) and group 3 (69 %). There were no differences in terms of reinterventions.

**Table 5:** Comparison of follow-up results in groups 1–3

	Group 1 (*n* = 75)	Group 2 (*n* = 80)	Group 3 (*n* = 129)
Amputation-free survival (%)			
At 6 months	93	77	80
At 12 months	86	65	69
Freedom from amputation (%)			
At 6 months	98	90	85
At 12 months	94	80	81
Survival (%)			
At 6 months	95	85	92
At 12 months	92	76	85
Freedom from reintervention (%)			
At 6 months	78	78	74
At 12 months	70	71	69

**Fig. 1:**
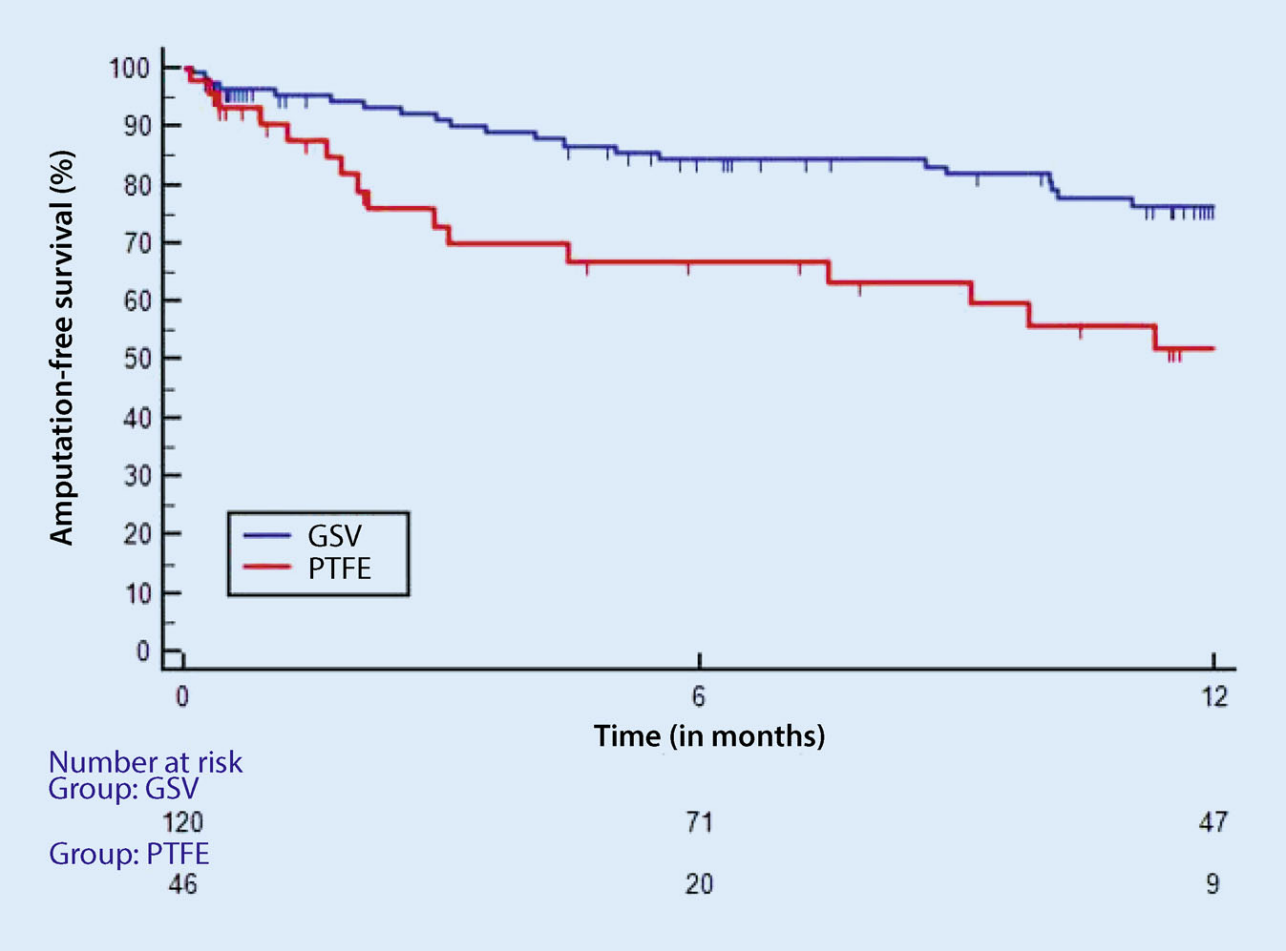
Amputation-free survival in CLI patients with vein grafts (great saphenous vein, GSV) vs. polytetrafluoroethylene (PTFE) grafts. Only bypasses with distal anastomoses below the knee. Hazard ratio (HR) 2.27, 95 % confidence interval (CI) 1.08–4.75, *p* = 0.009

Table 6 shows the results according to the bypass material used (i.e. vein vs. prosthetic graft). In the vein graft group, there were no differences in amputation-free survival at 1 year according to the site of distal anastomosis, whereas 1‑year results for the prosthetic grafts were significantly better in group 1 at 92 % (*p* = 0.002) compared with group 2 (amputation-free survival 54 %) and group 3 (56 %). In the above-knee bypasses, synthetic material was at least not inferior to vein bypasses (amputation-free survival: prosthetic graft 92 %, vein graft 71 %, *p* = 0.147), whereas in the crural/pedal bypasses, vein grafts showed better amputation-free survival at 1 year (76 %) compared with prosthetic grafts (56 %); however, even this difference was not statistically significant (*p* = 0.105). This statement and calculation (*p*-value 0.105) relates to the comparison of all vein grafts, i.e. upper extremity, great saphenous vein (GSV) and small saphenous vein with all prosthetic grafts, e.g. Dacron and polytetrafluoroethylene (PTFE). If GSV bypasses are compared only with PTFE prosthetic bypasses (Fig. 1), significantly better amputation-free survival can be observed for the former compared with the latter (*p* = 0.009) in the case of distal anastomoses.

**Table 6:** Follow-up outcomes in groups 1–3 (vein vs. prosthetic graft)

	Group 1 (*n* = 75)	Group 2 (*n* = 80)	Group 3 (*n* = 129)
*Vein graft, n*	27	46	86
Amputation-free survival (%)			
At 6 months	89	83	83
At 1 year	71	72	76
Freedom from intervention (%)			
At 6 months	81	74	80
At 1 year	61	67	74
*Prosthetic graft, n*	48	34	43
Amputation-free survival (%)			
At 6 months	92	69	74
At 1 year	92	54	56
Freedom from intervention (%)			
At 6 months	75	85	61
At 1 year	72	79	61

Table 7 provides a break-down of the follow-up results according to a number of risk factors. Patients in Rutherford stage 4 exhibited significantly better amputation-free survival at 1 year (78 %) compared with Rutherford stage 6 patients (61 %, *p* = 0.045). The differences in 1‑year results according to PIII CLI risk score were also significant (PIII CLI risk score ≤3: amputation-free survival 78 % vs. PIII CLI risk score 4–7: amputation-free survival 69 %, *p* = 0.053). The least favorable results were seen in the 11 patients with PIII CLI risk score ≥8, who exhibited an amputation-free survival of only 50 % at 1 year (*p* = 0.076). One striking feature was that there were no significant differences in 1‑year results (*p* = 0.693) regardless of whether no or three vessels were open (1-year amputation-free survival 74 % vs. 78 %) or whether diabetes was present or not (1-year amputation-free survival 69 % vs. 75 %, *p* = 0.486). Previous vascular interventions had no statistically significant effect on the results (1-year amputation-free survival 68 % vs. 77 % in the case of no previous intervention, *p* = 0.124).

**Table 7:** Follow-up outcomes according to CLI category, run-off and other risk factors

	Amputation-free survival (%) at 6 months	Amputation-free survival (%) at 1 year
Rutherford class 4, *n* = 72	91	78
Rutherford class 5, *n* = 140	78	74
Rutherford class 6, *n* = 72	80	61
Run-off, vessels open		
0, *n* = 31	81	74
1, *n* = 134	78	68
2, *n* = 76	91	80
3, *n* = 43	83	78
PIII CLI risk score ≤3, *n* = 135	88	78
PIII CLI risk score 4–7, *n* = 138	78	69
PIII CLI risk score ≥8, *n* = 11	75	50
Diabetics, *n* = 136	81	69
Non-diabetics, *n* = 148	85	75
Obesity (BMI >30 kg/m^2^), *n* = 40	81	72
BMI <30 kg/m^2^, *n* = 244	82	76
Previous vascular procedure		
Yes, *n* = 139	79	68
No, *n* = 145	86	77
Vein graft, *n* = 159	84	74
Prosthetic graft, *n* = 125	80	70

*BMI* body mass index, *PIII CLI* PREVENT III critical limb ischemia

## Discussion

The Vascular Study Group of New England database includes 2110 bypasses for CLI for the period 2003–2009 [7]. The mean patient age was 69.9 years, 5 years older than the mean age of the 797 claudication patients studied simultaneously. Some 24.7 % of CLI patients received a prosthetic graft. While hospital mortality was low (2.1 %) reoperations were required in 15 % of cases and wound infections were observed in 5.6 % of patients. During the 1‑year follow-up period 13.6 % of the patients died and the major amputation rate was 12.2 %. The primary graft patency rate was 66.4 % and the secondary patency rate was 77.4 %. Thus, the results for CLI were significantly less favorable compared with the intermittent claudication (IC) patients also investigated in the study. In the IC group 1‑year mortality was only 3.7 %, the major amputation rate 1.6 %, primary graft patency 78.9 % and secondary patency 89 % at 1 year. These results underline the impact of the baseline situation on the results of peripheral bypass surgery. As patients with CLI and IC have widely differing prognoses, the two groups need to be considered separately; this also applies to perioperative results. For CLI patients in the US nationwide inpatient sample (NIS), Sachs et al. [8] reported a hospital mortality of 2.6 % and a major amputation rate of 3.9 % following open bypass surgery, which is consistent with the results obtained in this study (hospital mortality in the total patient population for all bypasses 2.8 % and major amputation rate 3.5 %). Furthermore, the analysis conducted by Sachs et al. [8] showed the amputation rate to crucially depend on the stage of CLI. Patients with rest pain or ulcers exhibited a perioperative amputation rate following bypass surgery of only 1.0 % and 0.7 %, respectively, whereas a rate of 9.1 % was observed in patients with gangrene. We distinguished CLI according to the Rutherford categories. Patients with Rutherford grade 4 exhibited significantly fewer postoperative complications (complication rate 16.7 %) compared with Rutherford grade 6 patients (45.8 %) and only 69.4 % of bypasses were open on discharge in Rutherford grade 6 patients compared with 88.9 % in Rutherford grade 4 patients (Table 4), which was also a statistically significant difference. Similarly, run-off status had a significant effect on the immediate postoperative outcome: if no vessel was open, a graft patency rate of 64.5 % was seen at patient discharge, compared with 85.5 % in the case of run-off from at least one vessel. Similarly, an analysis of results performed by Hiramori et al. [9] of endovascular treatment for femoropopliteal lesions indicated that having any run-off at all is the key to a better outcome: the quality of the run-off is important, not the number of open vessels; however, it must be said that at 1‑year follow-up, amputation-free survival in the present study did not differ according to whether run-off was present at baseline or not (Table 7).

It is important to pose the question in this context as to whether previous ipsilateral vascular interventions had a negative impact on results. This was not the case as far as the immediate postoperative result was concerned: the graft patency rate in the present patient population on discharge was 80.6 % (previous vascular intervention) and 85.5 % (no previous vascular intervention); however, better amputation-free survival was seen at 1 year in those patients who had not undergone previous vascular interventions (77 % vs. 68 %). This result shows a trend towards being consistent with observations in the Vascular Study Group of New England registry for 2003–2009 in this respect [10]. In that particular study, no immediate postoperative differences following infrainguinal bypasses were seen between patients with and without previous vascular interventions; however, the major amputation rate at 1 year among patients with previous endovascular interventions was significantly higher at 31 % compared with patients without previous ipsilateral revascularization (20 %). This observation has not been confirmed by others. Santo et al. [11] described the long-term outcome of 314 peripheral autologous vein bypasses (60 % infrapopliteal) in CLI patients. The 30-day mortality rate was 3.5 % and 5‑year outcomes were primary patency rate 45 %, secondary patency rate 64 %, limb salvage 89 % and amputation-free survival 49 %. No significant differences in outcome were seen according to whether patients had undergone previous endovascular interventions or not. It should be emphasized, however, that the 37 patients in the BASIL trial who received a bypass after initially failed angioplasty had a significantly worse amputation-free survival (*p* = 0.006) and somewhat worse overall survival (not significant) compared with the 184 bypass surgery-first patients [12].

**Fig. 2:**
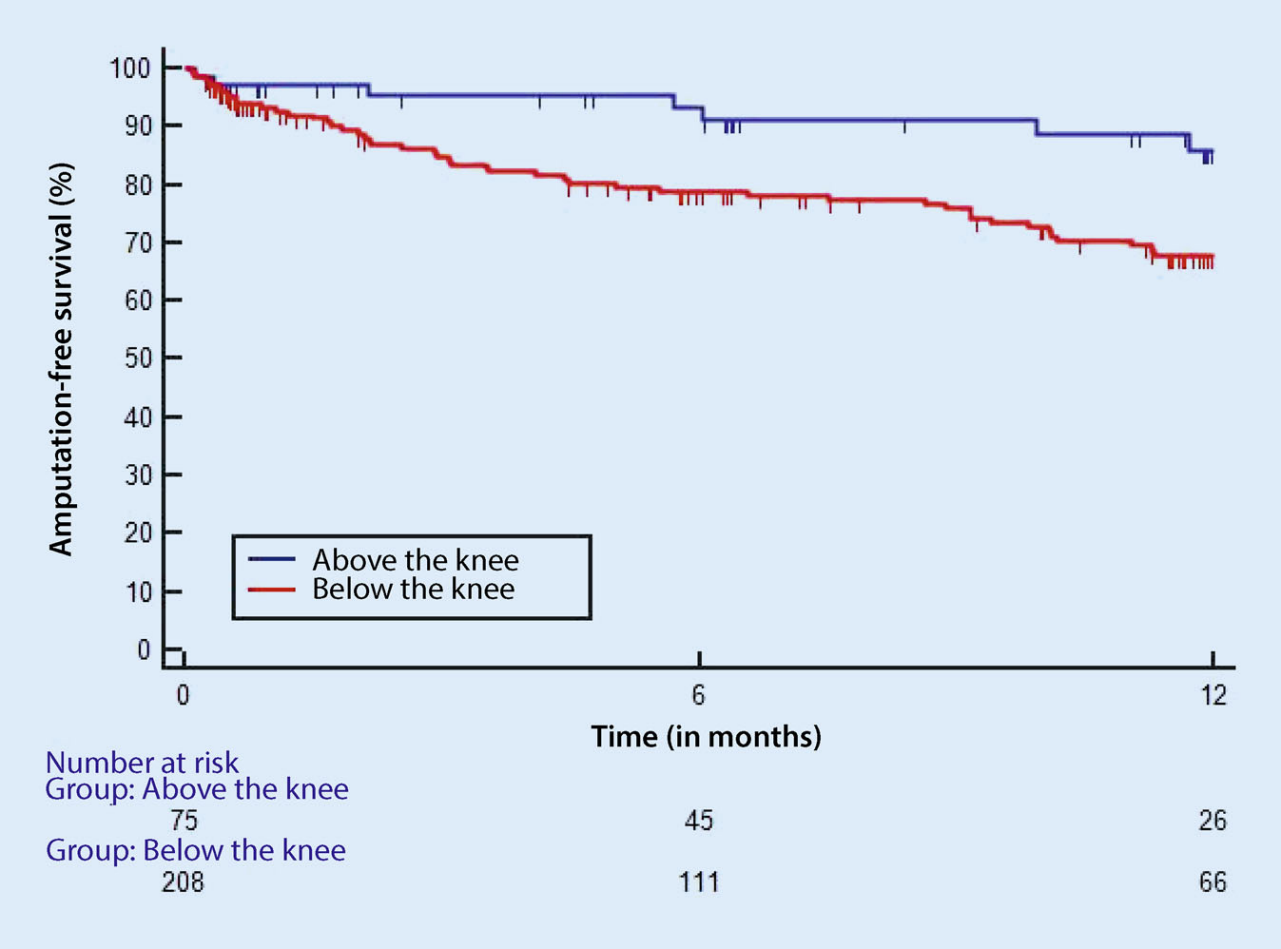
Amputation-free survival in CLI patients with bypasses above and below the knee (including crural and pedal targets). Hazard ratio (HR) 2.80, 95 % confidence interval (CI) 1.58–4.96, *p* = 0.008. *GSV* great saphenous vein, *PTFE* polytetrafluoroethylene

This study determined the PREVENT III (PIII) CLI risk score, a stratification model designed to estimate amputation-free survival in CLI and infrainguinal bypass surgery patients. As Table 4 shows, the PIII CLI risk score correlates only partially with the postoperative complication rate and although patients in the lowest score group had fewer complications compared with the others, this difference was not significant. There were also no differences in graft patency rates in the three risk groups on discharge; however, the lower the PIII CLI risk score, the better the amputation-free survival at 1‑year follow-up (Table 7). Mc Phee et al. [13] analyzed 30-day unplanned readmission following peripheral bypass surgery in 1543 patients, 84.5 % of whom had CLI. Readmission, among other factors, was significantly related to the level of the distal anastomosis (e.g. popliteal, crural or pedal) and the lower the anastomosis, the more frequent readmissions were. In turn, the readmission rate correlated long-term with the amputation rate. These results provide evidence that the outcome of peripheral bypass surgery depends on the level of peripheral anastomosis. This was also demonstrated by Suckow et al. [2]. Patients receiving a vein bypass to the popliteal artery below the knee showed a primary graft patency rate of 78 % (prosthetic graft 74 %). Outcomes of bypasses targeting crural/pedal vessels were significantly less favorable (vein graft patency rate 62 % and prosthetic graft 66 %). What was striking in this study was that there were no significant differences between vein and prosthetic grafts in terms of primary patency at 1 year; however, the major amputation rate when the popliteal artery was the target and a prosthetic graft was used was worse (15 %) compared with when a vein graft was used (8 %); however, in the case of more distal crural/pedal anastomosis, there were no differences in major amputation rates between vein and prosthetic grafts (major amputation rate at 1 year for prosthetic grafts 19 % and for vein graft, 14 %). In our own patient population, freedom from amputation and hence also amputation-free survival (Fig. 2) at 12 months, was significantly higher if the graft target was above the knee compared with below-knee targets; however, this statement is only valid if, as shown in Fig. 2, no distinction is made between the type of graft material. Indeed, no difference was seen between the vein graft groups, whereas amputation-free survival at 1 year among patients receiving prosthetic grafts to suprapopliteal targets was 92 % and to infrapopliteal and crural targets 54 % and 56 %, respectively (Table 6).

Vein grafts with infrapopliteal and crural targets therefore showed better outcomes than prosthetic grafts (Fig. 1). This is consistent with observations made in the retrospective analysis performed by Uhl et al. [14], who saw a primary patency rate of 68.2 % in the vein group and only 34.1 % in the heparin-bonded PTFE graft group at 3 years. The limb salvage rate was 81.8 % in the vein group vs. 56.5 % in the heparin-bonded PTFE graft group and patient survival was 62.8 % vs. 46.7 %, respectively. Neville et al. [15] also performed a retrospective comparison of heparin-bonded PTFE grafts (*n* = 62) and vein grafts (*n* = 50). Tibial artery bypasses were performed with heparin-PTFE grafts anastomosed using an autologous vein patch. The indication for surgery was CLI in the vast majority of cases (91.9 % in the PTFE group and 90 % in the vein group). At 86 %, the 1‑year primary vein graft patency rate was superior to that of PTFE grafts (75.4 %); however, there were no significant differences between the two groups in terms of amputation rates.

Some of the differences reported in the literature in results obtained from the comparison of vein vs. prosthetic grafts may be based on the fact that one study compared PTFE grafts and one study heparin-bonded PTFE grafts with vein grafts. Dorigo et al. [16] retrospectively evaluated results from an Italian multicenter registry and compared data on 180 below-knee bypasses using heparin-bonded PTFE prosthetic grafts in diabetic CLI patients with 133 autologous vein grafts for the same indication. They reported 30-day bypass patency rates for the 2 groups of 92.2 % and 93.2 %, respectively. Estimated patient survival rates at 48 months were 76.6 % (heparin-PTFE) vs. 72.7 % (vein). The primary graft patency rate was significantly better in the vein group (63.5 %) compared with the heparin-bonded PTFE-graft group (46.3 %). Differences in secondary graft patency (69.6 % vs. 57.5 %), limb salvage (82.4 % vs. 75.4 %), and amputation-free survival (64.4 % vs. 59.9 %) between vein and heparin-bonded PTFE grafts were not significant. Gessaroli et al. [17] undertook a comparison of vein vs. heparin-bonded grafts in a total of 74 femorocrural bypasses. This study found no differences in primary patency rates between the two graft materials (84 % for both groups) and the same applied to limb salvage rates (heparin-PTFE 87 % and vein 84 %) and 1‑year patient survival (87 % for both groups). Finally, Avgerinos et al. [18] reported on the retrospective analysis of a collective comprising a total of 407 infrainguinal bypasses including 116 popliteal (29 %), 226 tibial (56 %) and 65 pedal (16 %). Of these bypasses 96 % were in CLI patients whereby GSV grafts were performed in 63 % (*n* = 255), alternative autologous vein grafts (upper extremity veins and composite vein segments) in 26 % (106) and prosthetic grafts in 11 % (*n* = 47), of which 41 were heparin-bonded PTFE grafts. They reported a primary patency rate at 2 years of 47 % for the GSV bypasses, 24 % for the alternative veins and 43 % for the prosthetic bypasses. Secondary patency at 2 years was calculated at 75 %, 57 % and 46 %, respectively, for the three groups. In this particular investigation, using GSV was significantly superior in terms of secondary patency compared with the other materials used; however, there was no significant difference between the alternative vein grafts and prosthetic grafts. The 2‑year limb salvage rates were 86 %, 78 % and 72 %, respectively. These differences were not statistically significant. The key message of this investigation was that in the absence of the GSV as bypass material, complex reconstruction with alternative autologous vein grafts confers no advantages over heparin-bonded PTFE prosthetic grafts in below-knee bypasses. It should be noted that no distinction was made in our study between PTFE grafts with and without heparin bonding.

The present study had a number of limitations: study design was a retrospective analysis of prospectively recorded data, the study was not randomized and the number of patients in each group was small. In this respect it is not possible to exclude a type II statistical error in the analysis of the different endpoints between groups as well as in the subgroup analysis. The statistically significant differences should be considered with caution. Clinical evaluations of patients as well as angiographic and duplex ultrasound results were analyzed by the attending physician and the data entered in the registry. There was no core laboratory or adverse event committee. This registry reflects the results of the 27 participating centers; however, these are not necessarily representative of the situation in all vascular centers in Germany. Furthermore, it should be noted from a critical perspective that the results of revascularization also depend on anticoagulation and antiplatelet therapy. The present study was not able to analyze concomitant medication over time.

## Conclusion

- The present investigation demonstrated a trend, albeit not a statistically significant one, towards fewer complications in the immediate postoperative course in CLI patients treated with above-knee bypasses compared with more distal bypass targets. The same applied (with statistical significance) to Rutherford class 4 vs. Rutherford class 6 patients and to non-diabetics compared with diabetics. The greatest differences in terms of graft patency rates at the time of discharge were seen between Rutherford class 6 (69.4 % open) vs. Rutherford class 4 (88.9 % open) and run-off (no run-off vessel vs. one run-off vessel detected: bypass open in 64.5 % vs. 85.8 % of cases).
- At 1‑year follow-up, amputation-free survival was significantly better in patients with above-knee bypasses compared to bypasses with more distal targets; in patients with Rutherford class 4 vs. Rutherford class 6; and in patients with a PIII CLI risk score ≤3 vs. a PIII CLI risk score of 4–7 (*p* = 0.053).
- No differences were seen in amputation-free survival at 1 year according to the site of distal anastomosis in the vein graft group, whereas 1‑year outcomes for prosthetic bypasses were significantly better when targets were suprapopliteal compared with infrapopliteal and crural bypasses.
- In patients receiving above-knee bypasses, prosthetic grafts were at least not inferior to vein grafts, whereas in the group receiving crural or pedal bypasses, GSV grafts showed better amputation-free survival at 1 year compared with prosthetic grafts.
